# Drug repositioning for esophageal squamous cell carcinoma

**DOI:** 10.3389/fgene.2022.991842

**Published:** 2022-09-28

**Authors:** Adam N. Bennett, Rui Xuan Huang, Qian He, Nikki P. Lee, Wing-Kin Sung, Kei Hang Katie Chan

**Affiliations:** ^1^ Jockey Club College of Veterinary Medicine and Life Sciences, City University of Hong Kong, Kowloon, Hong Kong SAR, China; ^2^ Department of Electrical Engineering, City University of Hong Kong, Hong Kong, Hong Kong SAR, China; ^3^ Department of Biomedical Sciences, City University of Hong Kong, Hong Kong, Hong Kong SAR, China; ^4^ Department of Surgery, The University of Hong Kong, Pokfulam, Hong Kong SAR, China; ^5^ Department of Computer Sciences, National University of Singapore, Singapore, Singapore; ^6^ Department of Epidemiology, Centre for Global Cardiometabolic Health, Brown University, Providence, RI, United States

**Keywords:** drug repositioning, drug repurposing, esophageal squamous cell carcinoma, ESCC treatment, cancer biology

## Abstract

Esophageal cancer (EC) remains a significant challenge globally, having the 8th highest incidence and 6th highest mortality worldwide. Esophageal squamous cell carcinoma (ESCC) is the most common form of EC in Asia. Crucially, more than 90% of EC cases in China are ESCC. The high mortality rate of EC is likely due to the limited number of effective therapeutic options. To increase patient survival, novel therapeutic strategies for EC patients must be devised. Unfortunately, the development of novel drugs also presents its own significant challenges as most novel drugs do not make it to market due to lack of efficacy or safety concerns. A more time and cost-effective strategy is to identify existing drugs, that have already been approved for treatment of other diseases, which can be repurposed to treat EC patients, with drug repositioning. This can be achieved by comparing the gene expression profiles of disease-states with the effect on gene-expression by a given drug. In our analysis, we used previously published microarray data and identified 167 differentially expressed genes (DEGs). Using weighted key driver analysis, 39 key driver genes were then identified. These driver genes were then used in Overlap Analysis and Network Analysis in Pharmomics. By extracting drugs common to both analyses, 24 drugs are predicted to demonstrate therapeutic effect in EC patients. Several of which have already been shown to demonstrate a therapeutic effect in EC, most notably Doxorubicin, which is commonly used to treat EC patients, and Ixazomib, which was recently shown to induce apoptosis and supress growth of EC cell lines. Additionally, our analysis predicts multiple psychiatric drugs, including Venlafaxine, as repositioned drugs. This is in line with recent research which suggests that psychiatric drugs should be investigated for use in gastrointestinal cancers such as EC. Our study shows that a drug repositioning approach is a feasible strategy for identifying novel ESCC therapies and can also improve the understanding of the mechanisms underlying the drug targets.

## Introduction

**FIGURE 1:**
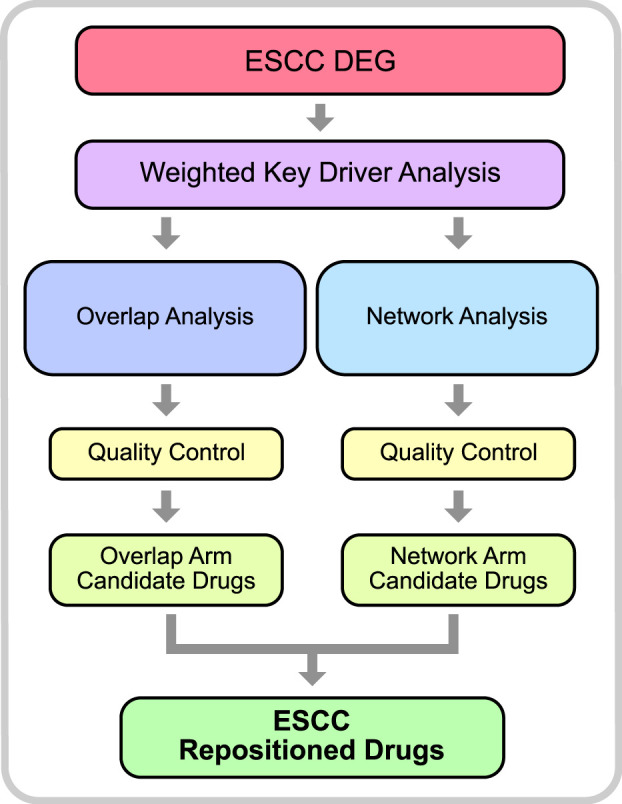
Drug Repositioning Analysis Methodology. The initial step of the analysis included differential gene expression on previously published array data from GEO (accession: GSE23400). DEGs were then used to identify key driver genes in a weighted key driver analysis. The key driver genes were then used as input in 2 arms; overlap analysis and network analysis. Qualify control was performed to filter out erroneous results and identify candidate drugs. Drugs which were common to both arms were considered robust and considered ESCC Repositioned Drugs.

There are two major subtypes of Esophageal cancer (EC), esophageal squamous cell carcinoma (ESCC) and esophageal adenocarcinoma (EAC) (Ye et al., 2021). In China, more than 90% of esophageal cancer cases are ESCC (Zhang X. et al., 2021). EC as a whole remains a significant challenge globally, having the 8^th^ highest incidence and the 6th highest mortality worldwide killing over 500,000 people in 2020 (Sung et al., 2021). A major driver of the high mortality rate is likely due to the fact that there are very few effective therapeutic options for EC patients. In recent years, there has been a significant increase in survival for many cancers, largely due to the availability of targeted therapies. For EC, however, targeted therapies are yet to make a significant impact on patient survival. Consequently, patients are often relying on more traditional therapies such as chemotherapy and surgical resection. In-order-to increase patient survival, novel therapeutic strategies for EC patients must be devised. Unfortunately, the development of novel drugs also presents its own significant challenges as most novel drugs do not make it to market due to lack of efficacy or safety concerns. Therefore, it is more time and cost effective to identify existing drugs, that have already been approved for treatment of other diseases, which can be repurposed to treat EC patients. This can be achieved using a drugs gene signature, the alterations in gene expression as a result of exposure to the drug. The gene signature of a drug indicates the underlying biological pathways and mechanisms that are involved in the therapeutic effect of the drug. With this knowledge, we can then identify candidate drugs which have gene signatures capable of reversing aberrant gene expression patterns observed in disease-states to those observed in normal cells. This gene signature-based approach has been adopted by previous research to identify drugs that can be repositioned to treat a variety of diseases including, but not limited to, cancer, Alzheimer’s, hyperlipidaemia, hypertension, and inflammatory disease (Corbett et al., 2012; Hall et al., 2014; Guney et al., 2016; Subramanian et al., 2017; Cheng et al., 2018; Carvalho et al., 2021; Wu et al., 2022). To date, drug repositioning to target gene signatures has primarily involved identifying directly overlapping drug genes and disease genes (herein referred to as overlap analysis) (Subramanian et al., 2017; Wang et al., 2018; Chen et al., 2022). More recently, network analysis has been greatly employed in this area as it offers distinct advantages over more traditional statistical methods. This is due to the fact that the models that can be built with this methodology are an excellent way to capture a molecules relationship with other molecules. In particular, nodes can be used to represent multiple entities such as genes, molecules, proteins, etc, and the edges can also represent a vast array of information such as mode-of-actions (MoAs), underlying mechanisms, or functional similarities (Jarada et al., 2020) Hence, network-based methods can accurately represent the biological mechanisms which are driving diseases (Barabási et al., 2011). As a result, network-based drug repositioning can identify drugs which target the underlying biology of the disease. It is worth noting, however, that other methods of computational drug repositioning have also been adopted, such as Data Mining and Machine Learning. An excellent review of the different methodologies, as well as their advantages and disadvantages has recently been published (Jarada et al., 2020). Due to the success of drug repositioning overall, and the absence of effective treatments for ESCC, it has been proposed that this method be used to identify novel treatment strategies for ESCC. However, these studies have largely, though not completely, been limited to testing existing cancer drugs *in vitro* with drug screening methods (Xie et al., 2020; Li Y. et al., 2021). Herein, we adopt both a network-based and overlap-based drug repositioning methodology, to identify existing drugs that can specifically target the aberrant expression profile of ESCC and impede oncogenesis. To do this, we used previously published data for *in-silico* drug repositioning analysis utilising the PharmOmics webserver (Chen et al., 2022). The repositioning analysis consisted of two arms, the ‘overlap analysis’ arm and the ‘network analysis’ arm (Figure 1), which utilise two methods of drug repositioning.

## Results

### Identification of DEGs

The dataset GSE23400 was downloaded using the GEOquery R package function getGEO. In total, 167 DEGs were identified between ESC and normal samples (Details of the differential gene expression analysis can be found in the methods section). Of which, 65 were upregulated and 102 were downregulated. The top 5 most upregulated genes are *MMP1, SPP1, POSTN, COL1A1,* and *JUP.* The top 5 most downregulated genes are *CRISP3*, *MAL*, *CRNN*, *SCEL*, *CLCA4*. The top 25 up-regulated genes can be observed in Table 1, whereas the top 25 down-regulated genes can be observed in Table 2.

**TABLE 1:** Top 25 up-regulated genes in differential gene expression analysis comparing cancer tissue with adjacent tissue in ESCC patients.

Gene	logFC	Adj. P-value
MMP1	4.443	8.65 × 10^–29^
SPP1	3.187	3.38 × 10^–23^
POSTN	3.066	2.03 × 10^–22^
COL1A1	2.990	5.89 × 10^–32^
JUP	2.831	1.68 × 10^–16^
COL1A2	2.698	2.95 × 10^–26^
COL11A1	2.405	1.64 × 10^–20^
CDH11	2.370	1.14 × 10^–21^
MMP12	2.240	5.17 × 10^–19^
MAGEA6	2.226	2.40 × 10^–09^
PTHLH	2.213	7.27 × 10^–13^
MAGEA3	2.213	1.71 × 10^–09^
VCAN	2.204	2.11 × 10^–20^
SNAI2	2.202	2.62 × 10^–25^
MMP10	2.193	8.12 × 10^–11^
COL3A1	2.164	7.24 × 10^–22^
SULF1	2.125	1.69 × 10^–22^
ECT2	2.112	2.30 × 10^–31^
COL5A2	2.087	1.59 × 10^–20^
TOP2A	2.004	2.93 × 10^–23^
PLAU	1.994	4.17 × 10^–27^
CKS2	1.968	1.90 × 10^–22^
INHBA	1.904	2.28 × 10^–15^
ISG15	1.870	8.29 × 10^–14^
CEP55	1.846	5.48 × 10^–26^

**TABLE 2:** Top 25 down-regulated genes in differential gene expression analysis comparing cancer tissue with adjacent tissue in ESCC patients.

Gene	logFC	Adj. P-value
CRISP3	−4.247	8.53 × 10^–21^
MAL	−3.968	8.65 × 10^–20^
CRNN	−3.654	3.31 × 10^–16^
SCEL	−3.496	4.16 × 10^–17^
CLCA4	−3.425	2.59 × 10^–18^
TGM3	−3.329	5.45 × 10^–19^
CRCT1	−3.175	2.76 × 10^–15^
TMPRSS11E	−3.106	3.69 × 10^–15^
SLURP1	−2.952	1.03 × 10^–17^
CLIC3	−2.913	7.72 × 10^–17^
ENDOU	−2.774	3.52 × 10^–21^
IL1RN	−2.769	2.06 × 10^–22^
PPP1R3C	−2.750	5.02 × 10^–24^
SPINK5	−2.745	8.77 × 10^–17^
HPGD	−2.647	5.22 × 10^–24^
RHCG	−2.628	7.00 × 10^–13^
KRT4	−2.606	5.59 × 10^–14^
FLG	−2.432	2.72 × 10^–15^
KLK13	−2.353	1.73 × 10^–20^
ECM1	−2.351	8.47 × 10^–17^
KRT13	−2.305	3.20 × 10^–10^
CEACAM6	−2.291	8.44 × 10^–13^
ADH1B	−2.288	3.47 × 10^–20^
PSCA	−2.260	2.25 × 10–^15^
HOPX	−2.233	7.07 × 10^–15^

### Functional and pathway enrichment analyses

Functional and pathway enrichment analyses were performed used the ‘clusterProfiler’ R package. Gene Set Enrichment Analysis (GSEA) was performed with Gene Ontology (GO) (hereafter referred to as GSEA-GO) and Kyoto Encyclopaedia of Genes and Genomes (KEGG) pathway (hereafter referred to as GSEA-KEGG). GSEA-GO analysis was performed with the gene set categories Biological Process (BP), Cellular Component (CC), and Molecular Function (MF), which identified 253, 36, 25 enriched gene sets, respectively. Numerous BP gene sets identified by the analysis are related to extracellular matrix and cell differentiation. Ranking BP analysis by adjusted p-value, the top 5 most enriched gene sets are cellular component organization, cellular component organization or biogenesis, extracellular matrix organization, extracellular structure organization, multicellular organism development. According to adjusted p-value, the top 5 most enriched CC category are endoplasmic reticulum lumen, external encapsulating structure, extracellular matrix, fibrillar collagen trimer, banded collagen fibril. Furthermore, the top 5 categories in the MF analysis identified extracellular matrix structural constituent, extracellular matrix structural constituent conferring tensile strength, protein-containing complex binding, cell adhesion molecule binding, glycosaminoglycan binding. The full results for BP, CC, MF can be observed in Supplementary Tables S1–S3, respectively. Gene Set Enrichment Analysis of KEGG (GSEA-KEGG) identified 12 enriched gene sets (Supplementary Table S4), including those previously identified as ESCC-related, such as Focal adhesion, ECM-receptor interaction, PI3K-Akt signalling pathway.

### Weighted key driver analysis

Weighted Key Driver Analysis (wKDA) was performed using Mergeomics webserver. In this analysis, genes which possess a local network neighbourhood that have a significant enrichment of genes that are ESCC-associated are considered key drivers (KDs) (Ding et al., 2021). The analysis identified 89 key driver genes which were then filtered to select those which possessed an FDR <0.05, ensuring that only the strongest KDs are used in subsequent analyses. This resulted in 39 key driver genes (Supplementary Table S5). The top 10 key driver genes are *NCAPG*, *PLG*, *NUSAP1*, *COL17A1*, *ASPM*, *TOP2A*, *ITGB3*, *P4HB*, *TTK*, and *COL7A1*.

### Repositioned drugs

Drug repositioning analysis was performed using both the Overlap Drug Repositioning and the Network Drug Repositioning modules from PharmOmics (Chen et al., 2022). The potential drugs from the analysis, were then filtered to identify robust ESCC repositioned drugs. The repositioning analysis identified 25 drugs that are strong candidates for ESCC treatment (Table 3). The top 10 repositioned drugs are Erlotinib, Palbociclib, Doxorubicin, Methotrexate, Crizotinib, Vinblastine, Gemcitabine, Daunorubicin, Venlafaxine, and Ethanol. We predicted that drugs which interact with EGFR (Erlotinib, Crizotinib, and Lapatinib), estrogen signalling (Tamoxifen, Fulvestrant, Hydrocortisone, and Anastrozole) and TRAIL-mediated apoptosis (Azithromycin and Anastrozole) pathways have potential for treating ESCC.

**TABLE 3:** ESCC repositioned drugs.

Drug	Study	z-score	Jaccard score	Odds ratio	Adj. P-value	Within species rank
Erlotinib	*In Vitro*	−8.806362317	1.59 × 10^–2^	2.16 × 10^1^	5.66 × 10^–5^	0.956
Palbociclib	*In Vitro*	−8.090451972	1.56 × 10^–2^	2.11 × 10^1^	6.18 × 10^–5^	0.953
Doxorubicin	*In Vitro*	−7.851164741	3.41 × 10^–2^	5.35 × 10^1^	5.13 × 10^–9^	0.993
Methotrexate	PharmOmics meta	−7.504929239	1.35 × 10^–2^	1.83 × 10^1^	7.80 × 10^–4^	0.930
Crizotinib	*In Vitro*	−7.50277149	2.08 × 10^–2^	2.95 × 10^1^	1.61 × 10^–6^	0.980
Vinblastine	PharmOmics meta	−6.871294272	5.14 × 10^–2^	9.04 × 10^1^	2.10 × 10^–17^	0.998
Gemcitabine	*In Vitro*	−5.585120295	2.43 × 10^–2^	3.58 × 10^1^	3.91 × 10^–10^	0.987
Daunorubicin	*In Vitro*	−5.155171903	2.94 × 10^–2^	4.53 × 10^1^	2.07 × 10^–7^	0.991
Venlafaxine	*In Vitro*	−5.053770712	1.53 × 10^–2^	2.07 × 10^1^	6.74 × 10^–5^	0.950
Ethanol	PharmOmics meta	−4.264304125	2.33 × 10^–2^	3.41 × 10^1^	5.61 × 10^–10^	0.985
Tamoxifen	PharmOmics meta	−4.072907051	1.79 × 10^–2^	2.51 × 10^1^	3.24 × 10^–5^	0.969
Arsenic trioxide	PharmOmics meta	−3.980019706	4.67 × 10^–2^	7.95 × 10^1^	2.10 × 10^–11^	0.997
Dasatinib	*In Vitro*	−3.747277559	2.08 × 10^–2^	2.95 × 10^1^	1.61 × 10^–6^	0.980
Ixazomib	PharmOmics meta	−3.730099165	5.73 × 10^–2^	1.07 × 10^2^	5.33 × 10^–21^	0.999
Penicillamine	PharmOmics meta	−3.248848376	4.13 × 10^–2^	6.75 × 10^1^	1.53 × 10^–13^	0.996
Nefazodone	*In Vitro*	−3.176922914	1.15 × 10^–2^	1.51 × 10^1^	1.35 × 10^–3^	0.893
Leflunomide	PharmOmics meta	−2.888272698	4.65 × 10^–2^	7.91 × 10^1^	1.83 × 10^–15^	0.997
Fulvestrant	*In Vitro*	−2.792994137	2.35 × 10^–2^	3.41 × 10^1^	8.07 × 10^–7^	0.985
Azithromycin	*In Vitro*	−2.53558291	2.79 × 10^–2^	4.16 × 10^1^	2.17 × 10^–8^	0.990
Hydrocortisone	PharmOmics meta	−2.37861135	3.20 × 10^–2^	4.89 × 10^1^	5.37 × 10^–10^	0.992
Etanercept	PharmOmics meta	−2.333538798	1.50 × 10^–2^	2.32 × 10^1^	3.88 × 10^–3^	0.948
Acetaminophen	PharmOmics meta	−2.196753074	3.65 × 10^–2^	5.90 × 10^1^	2.93 × 10^–14^	0.994
Lapatinib	*In Vitro*	−2.141804897	2.63 × 10^–2^	3.93 × 10^1^	4.09 × 10^–7^	0.989
Niacin	PharmOmics meta	−2.092485943	2.25 × 10^–2^	3.24 × 10^1^	1.03 × 10^–6^	0.983
Anastrozole	PharmOmics meta	−2.073331977	3.75 × 10^–2^	6.08 × 10^1^	2.19 × 10^–14^	0.994

### Drug validation

To validate our findings, we performed a literature search to determine whether any of the drugs identified by our analysis are currently used in ESCC treatment (Table 4). We found that 7 of the top 10 repositioned drugs, according to z-score, are already used to treat ESCC or have been shown to demonstrate efficacy in clinical trials. Candidate drugs were then validated using Binding DB (Gilson et al., 2016). Each drug was searched in the database to ascertain whether they bind to proteins known to be involved in ESCC. We found that 21 out of 25 repositioned drugs have a strong binding affinity to proteins that have been associated with ESCC in some manner previously (Table 5). Additionally, we performed a literature search to assess whether there is any biological evidence (*in vitro* or *in vivo*) that demonstrates efficacy or establishes a plausible mechanism by which the novel repositioned drugs could be beneficial for ESCC patients (Table 6). We found that all of our novel ESCC drugs, except for Venlafaxine, target pathways or proteins which have been demonstrated to drive oncogenesis in several cancers, including ESCC. Therefore, these drugs should be able to target the underlying biological processes driving oncogenesis in ESCC and inhibit proliferation and/or initiate apoptosis in ESCC.

**TABLE 4:** Current use of ESCC Repositioned Drugs.

Drug	Standard treatment for ESCC/Clinical trial	Clinical trial remarks	Reference
Erlotinib	Yes	Limited activity in EC overall but response was observed in ESCC (Only 2/13 participants were ESCC)	Ilson et al. (2011)
		Promising results if combined with radiotherapy	Zhao et al. (2016)
Palbociclib	Yes	Not promising result in clinic trials. However, authors claim that the drug could be useful in combination with other drugs	Karasic et al. (2020)
Doxorubicin	Yes	Used successfully in combination with other drugs (cisplatin and fluorouracil combination therapy)	Honda et al. (2010)
Methotrexate	Yes	Used for palliative care in combination with other drugs	DUSI et al. (2020)
Crizotinib	No	—	—
Vinblastine	Yes	Phase 2 Clinical Trial - Promising results	Conroy et al. (1996)
Gemcitabine	Yes	Phase 1 Clinical Trial - Promising results	Oettle et al. (2002)
Daunorubicin	No	—	—
Venlafaxine	No	—	—
Ethanol	Yes	Used for palliative care. Evidence of use for unresectable in case report with combination with chemotherapy	Wadleigh et al. (2006)
Tamoxifen	No	—	—
Arsenic trioxide	No	—	—
Dasatinib	No	—	—
Ixazomib	No	—	—
Penicillamine	No	—	—
Nefazodone	No	—	—
Leflunomide	No	—	—
Fulvestrant	No	—	—
Azithromycin	No	—	—
Hydrocortisone	No	—	—
Etanercept	No	—	—
Acetaminophen	No	—	—
Lapatinib	No	—	—
Niacin	No	—	—
Anastrozole	No	—	—

**TABLE 5:** Binding DB Target Validation. Repositioned drugs were investigated using Binding DB to determine whether the proteins that the drugs have strong affinity to have been previously shown to be associated with ESCC.

Drug	Protein binding in homo sapiens	Binding protein ESCC-Associated	Reference
Erlotinib	Epidermal growth factor receptor (EGFR)	Yes	Kashyap and Abdel-Rahman, (2018)
Palbociclib	CDK9	Yes	Tong et al. (2017)
	CDK1	Yes	Zhang et al. (2021a)
	CDK2	Yes	Zhou et al. (2021)
	CDK4	Yes	Huang et al. (2021)
Doxorubicin	Androgen Receptor	Yes	Sukocheva et al. (2015)
Methotrexate	Dihydrofolate reductase	Yes - Indirectly through MDM2	Maguire et al. (2008)
	MMP7	Yes	Tanioka et al. (2003)
Crizotinib	Epidermal growth factor receptor (EGFR)	Yes	Kashyap and Abdel-Rahman, (2018)
	FLT3	Yes	Zhu et al. (2021)
Vinblastine	—	—	—
Gemcitabine	Equilibrative nucleoside transporter 1	Yes - Indirectly through mIR-1269	Xie et al. (2022)
Daunorubicin	Multidrug resistance protein 1	Yes	Zhang et al. (2016)
Venlafaxine	Sodium-dependent dopamine transporter	Yes	Guo et al. (2018)
Ethanol	—	—	—
Tamoxifen	17-beta-hydroxysteroid dehydrogenase type 3	No	—
Arsenic trioxide	—	—	—
Dasatinib	Tyrosine- and threonine-specific cdc2-inhibitory kinase	Yes (and also via CDK1)	Zhang et al. (2019)
Ixazomib	Proteasome component C5	No	—
Penicillamine	Bile salt export pump	Yes	Bernstein et al. (2009)
Nefazodone	Alpha-1A adrenergic receptor	Yes	Zhang et al. (2018)
	5-hydroxytryptamine receptor 2A	Yes	Wei et al. (2022)
Leflunomide	matrix metalloproteinase 1	Yes	Pang et al. (2016)
	Dihydroorotate dehydrogenase	Yes	Qian et al. (2020)
Fulvestrant	Estrogen receptor	Yes	Zhang et al. (2017)
Azithromycin	Cytochrome P450 3A4	Yes	Bergheim et al. (2007)
Hydrocortisone	Corticosteroid-binding globulin (SERPINA6)	Yes	Ma et al. (2019)
Etanercept	—	—	—
Acetaminophen	Carbonic anhydrase 12	Yes	Ochi et al. (2015)
	Dipeptidyl peptidase 3	Yes	Liu et al. (2021)
Lapatinib	Epidermal growth factor receptor (EGFR)	Yes	Kashyap and Abdel-Rahman, (2018)
	Receptor tyrosine-protein kinase erbB-2 (HER2 or ERBB2)	Yes	Rong et al. (2020)
Niacin	Hydroxycarboxylic acid receptor 2	No	—
	Xanthine dehydrogenase/oxidase	Yes	Li et al. (2021a)
Anastrozole	Cytochrome P450 19A1	Yes	Bergheim et al. (2007)

**TABLE 6:** Potential mechanism of action for novel drugs.

Drug	Potential mechanism of action	Additional remarks	Citation(s)
Crizotinib	Protein kinase inhibitor (inc. HGFR)	Acts as an inhibitor against anaplastic lymphoma kinase. Crizotinib is an inhibitor of c-Met and could be used to target HGF pathway	Digklia and Voutsadakis, (2013)
Daunorubicin	Intercalates with DNA and interrupts cell proliferation	—	Niaki et al. (2020)
Venlafaxine	—	Has been used for managing hot flashes during breast cancer therapy	Biglia et al. (2005)
Tamoxifen	Selective estrogen receptor modulator (SERM)/partial agonist of ER	Evidence of efficacy in cell and animal models. Preliminary evidence in adenocarcinoma of enhancing chemo therapy effect	Due et al., (2016); Huang et al., 2019; Wang et al., 2020
Arsenic trioxide	Induces programmed cell death	Evidence of DNA damage-mediated cyclin D1 degradation in ESCC cell lines	Zhu et al. (2020)
Dasatinib	Tyrosine kinase inhibitor	Dasatinib increases ESCC cell lines sensitivity to cisplatin	Chen et al. (2015)
Ixazomib	Inhibits the protein proteasome subunit beta type-5 (PSMB5)	Supresses proliferation in Esophageal squamous cell carcinoma in cell lines through c-Myc/NOXA pathway. *In vivo* evidence of efficacy in non-small cell lung cancer	Chattopadhyay et al., 2015; Wang et al., 2021b
Penicillamine	Radio-chemo-sensitisation involving H_2_O_2_-mediated oxidative stress	Enhances breast and lung cancer response to radiation and carboplatin via H_2_O_2_-mediated oxidative stress	Sciegienka et al. (2017)
Nefazodone	Disrupts mitochondrial function	Demonstrates anticancer properties in multiple cell lines	Varalda et al. (2020)
Leflunomide	Dihydroorotase dehydrogenase (DHODH) and/or Tyrosine kinase inhibition	Potential anticancer drug through disruption of pyrimidine synthesis and EGFR signalling. *In vitro* and *in vivo* evidence for inducing apoptosis in neuroblastoma	Zhu et al., 2013; Zhang and Chu, (2018)
Fulvestrant	Estrogen receptor antagonist	Results in complete inhibition of estrogen signalling through the ER	Nathan and Schmid, (2017)
Azithromycin	Apoptosis induction via TRAIL	Efficacy *in vitro* and *in vivo* in colon cancer by TRAIL autophagy	Qiao et al. (2018)
Hydrocortisone	Binds glucocorticoid receptor to inhibit inflammatory transcription factors	Evidence to suggest BRCA1 downregulation in breast cancer	Antonova and Mueller, (2008)
Etanercept	Tumour necrosis factor (TNF) inhibitor	Prolonged disease stabilisation was observed in EC used in combination with chemotherapy	Monk et al., 2006; Shirmohammadi et al., 2020
Acetaminophen	Apoptosis induction	Promising results used in combination with chemotherapy in lung cancer	Lee et al. (2019)
Lapatinib	tyrosine kinase inhibitor/EGFR/HER1 and HER2 receptors	ESCC cell and patient-derived xenograft model	HOU et al., 2013; Saito et al., 2015
Niacin	Modulation of NAD + levels	Evidence of TRAIL mediated autophagy in colon cancer	Kim et al. (2015)
Anastrozole	Aromatase Inhibition	Has been used with Anti-Fibroblast growth factor receptor 1 (FGFR1) drug in breast cancer. Evidence that FGFR1 can be used as a independent prognosis marker in ESCC and anti-FGFR1 decreases proliferation via MEK-ERK downstream pathways	Milani et al., 2009; Chen et al., 2017

## Discussion

ESCC is one of the most common malignancies and possess a significant mortality rate worldwide. This is largely due to late diagnosis and scarcity of efficacious treatment strategies upon being diagnosed (Feng et al., 2021). To address this, we performed a disease-based drug repositioning analysis with previously published ESCC gene expression data from paired patient samples. Differential gene expression analysis data identified 167 differentially expressed genes (DEGs) which were then used in wKDA and identified 39 key driver genes (KDGs). The genes with the highest absolute logFC identified by our differential gene expression analysis are *MMP1*, *CRISP3*, *MAL*, *CRNN*, *SCEL*. The most upregulated gene, *MMP1*, encodes a protein involved in the breakdown of the extracellular matrix (ECM) by cleaving collagens and other molecules. The most downregulated gene, *CRISP3*, encodes a protein located in the ECM and thought to be involved in cellular matrix remodelling (Ribeiro et al., 2011). The wKDA identified 39 significant driver genes for ESCC. Amongst the top 10 most significant KDGs, *NUSAP1*, *COL17A1*, *ITGB3* and *COL7A1* are involved in ECM maintenance. For example, the 4^th^ most significant key driver gene, *COL17A1*, encodes a protein involved in cell-matrix adhesion (Jones et al., 2020). Taken together, these results suggest that alterations in ECM are an important driver of ESCC oncogenesis (Chen et al., 2016). Furthermore, KEGG analysis found both Focal adhesion and ECM-receptor interaction to be the 3rd and 4th most enriched term, respectively. This is in line with previous research that indicates higher levels of Serum human relaxin 2 (H2 RLN), a protein involved in ECM, collagen, and matrix metalloproteinase is associated with worse prognosis, including higher clinical stage and poorer survival (Ren et al., 2013; Napier et al., 2014).

Using 39 KDGs in an ESCC drug repositioning analysis, we identified 25 drugs that are predicted to have therapeutic effect in ESCC. Of which, 7 are either currently used in the clinic or have been used in clinical trials and 2 have shown efficacy *in vitro* or *in vivo*. Importantly, those which have been used in clinical trials have demonstrated efficacy particularly when used in combination with other drugs, such as chemotherapy. This is not surprising, however, as combination therapy has long been a standard practice in cancer therapy, including for ESCC where the current first-line treatment regimen is a combination of 5-fluorouracil and cisplatin (Hiramoto et al., 2018; Hirano and Kato, 2019). Each repositioned drug was validated *in-silico* using the drug binding database BindingDB, to identify which drug targets have previously been associated with ESCC (Table 5). Significantly, 21 of the 25 repositioned drugs have targets that have previously been associated with ESCC in some manner, which demonstrate the robustness of our findings. To further validate our findings, we performed a literature search on the novel repositioned drugs to examine whether there is an underlying biological mechanism which would justify the drugs appearance in the results (Table 6). We found that almost all of the repositioned drugs have been shown to demonstrate anti-cancer effects in multiple cancers, most notably breast cancers and non-small cell lung carcinoma (NSCLC). Interestingly, many of the repositioned drugs target specific pathways; EGFR (Erlotinib, Crizotinib, and Lapatinib), estrogen signalling (Tamoxifen, Fulvestrant, Hydrocortisone, and Anastrozole) and TRAIL-mediated apoptosis (Azithromycin and Anastrozole) pathways, suggesting that these pathways are key drivers of ESCC. This is in line with previous research which identified the EGFR AND ER pathways as drivers of ESCC oncogenesis and metastasis and have also been associated with patient outcome (Maron et al., 2020). Crucially, some of these drugs have been shown to have therapeutic potential *in vitro*. For example, Lapatinib, which acts through EGFR and HER2 has been shown to be efficacious in ESCC patient-derived xenografts (Rong et al., 2020). The potential mechanisms by which novel drugs identified by our study can be observed in Table 6. It is also worth noting that there are 2 anti-depressants present in our results, Venlafaxine and Nefazodone. These results are particularly interesting as it has recently been shown that psychiatric drugs offer potential as anti-cancer therapeutics (Loehr et al., 2021). Moreover, a recent review has specifically addressed the need to investigate psychiatric drugs for treatment of gastrointestinal cancers (Avendaño-Félix et al., 2020). We hypothesise that Crizotinib, Lapatinib, and Dasatinib are amongst the drugs with the most potential. Particularly Crizotinib and Lapatinib are of note as they target the EGFR pathway which is already targeted in ESCC treatment with Erlotinib. Dasatinib also has high potential due to targeting Tyrosine- and threonine-specific cdc2-inhibitory kinase and CDK1, proteins known to be involved in ESCC, and also due to displaying efficacy in cell lines (Chen et al., 2015; Zhang et al., 2019).

There are several limitations to our study, however, most notably that due to limited data availability, the sample size of patient samples was relatively small. We were unable to stratify patients according to subtype of ESCC. This means that the analysis is focussed on ESCC as whole and does not take into consideration specific subtypes. Moreover, as multiple drugs identified in our study are more efficacious when in combination with another drug, it would be beneficial to know what other drugs should be used in combination with the novel therapeutics identified. However, this analysis does not predict drug combinations that would be effective in treating ESCC.

On the other hand, our study has several strengths. To our knowledge, this is the first study to adopt a primarily computational approach to perform drug repositioning analysis in ESCC. Particularly, there are studies that have a computational component, but they do not use patient samples to identify drugs based on network analysis of differentially expressed genes (Li et al., 2022). Moreover, this is also the first to adopt Pharmomics unique network analysis to perform the analysis on ESCC. Furthermore, as Pharmomics contains >18000 species/tissue-specific gene signatures for 941 drugs and chemicals, it provides a larger scope of potential drugs compared to other studies in ESCC. Another strength of the study is that we used a two armed approach to ensure robust findings as each repositioning methodology has its own strengths. The overlap-based repositioning allows us to identify drugs which target the KDGs whereas the network-based repositioning allows for insights into the molecular and mechanistic therapeutic effects of the drugs. As this specific form of network-based repositioning is unique to PharmOmics, our study can provide valuable insights into the underlying molecular mechanisms driving ESCC. Another strength of our study is the consistency of our results with previously published literature. DEGs which displayed the highest absolute logFC were consistent with previously published literature including *MMP1*, *SPP1*, *COL1A2*, and *COL1A1* amongst the top upregulated genes and *CRISPR3*, *MAL*, *TMPRSS11E*, and *CRNN* amongst the top downregulated genes (Feng et al., 2021; Song et al., 2021). Indeed, the gene with the highest absolute logFC, *MMP1*, is already known to be associated with ESCC oncogenesis (Chen et al., 2016). Additionally, higher *MMP1* is associated with poorer prognosis (Feng et al., 2021). Moreover, the most significant key driver genes (KDGs) identified by our wKDA are consistent with previously published studies (Li et al., 2020; Yu-jing et al., 2020; Wang M. et al., 2021). Significantly, multiple drugs identified by our analysis target key pathways known to be involved in ESCC oncogenesis and metastasis.

## Conclusion

Herein we utilised *in silico* disease-based drug repositioning to identify novel therapeutics for esophageal squamous cell carcinoma. Amongst 25 potential repositioned drugs identified in our study, 9 are currently used in the clinic or have shown promising results in clinical trials in combination with other treatments. Crucially, we identified 16 novel therapeutic strategies which possess a strong biological rationale for use in ESCC patients. Our study shows that drug repositioning approach is a feasible strategy in ESCC therapies and can improve the understanding of the mechanisms of the drug targets.

## Materials and methodology

### Data acquisition and identification of DEGs

Dataset was acquired from the Gene Expression Omnibus under accession code GSE23400 using the GEOquery R package function getGEO. The dataset consists of 53 paired patient samples from Esophageal squamous cell carcinoma (ESCC) patients. Additionally, 14,335 genes were in the dataset. Differentially expressed genes (DEGs) between paired tumour and non-tumour samples were identified using the limma package. The log-fold change (logFC) was calculated for DEGs. Genes with absolute logFC >1.5 and adjusted p-value < 0.01 were considered significant and used in subsequent analyses.

### Functional and pathway enrichment analyses

The DEGs identified above were analysed using the clusterProfiler R package in order to identify biological annotations from the Gene ontology (GO) functional enrichment and Kyoto Encyclopaedia of Genes and Genomes (KEGG). The GO analysis was performed for biological process (BP), cellular component (CC) and molecular function (MF). An adjusted p-value < 0.05 was considered as statically significant for all analyses.

### Weighted key driver analysis

Weighted key driver analysis (wKDA) was performed on DEGs using Mergeomics webserver to identify key driver genes (KDGs). wKDA has higher accuracy than standard key driver analysis as it considers edge weight information. The network used in the analysis was STRING PPI Network and default parameters were used (Search depth of 1, Undirected Edges, Min Hub Overlap of 0.33, and edge factor of 0.0). Genes which had an FDR <0.05 were considered as significant KDGs and used in subsequent analyses.

### Drug repositioning analysis

By the Pharmomics webserver, Drug Repositioning Analyses was performed using genes obtained from the wKDA analysis. The analysis consisted of two arms: the Overlap Drug Repositioning and ADR Analysis (Overlap-DR) arm and the Meta-Signature Network Drug Repositioning and ADR Analysis (Meta-Net-DR) arm. The network analysis adopted by Pharmomics uses a network proximity measure between drug DEGs and disease-related genes that has been adopted previously for protein-network-based analysis. Specifically, tissue-specific Bayesian gene regulatory networks (BNs) are used and then the mean shortest distance between drug DEGs and disease genes are tested. Hence, it combines species and tissue specific *in vivo* drug signatures with gene networks to identify connections between disease genes and known drug targets. On the other hand, overlap analysis adopted by Pharmomics is largely similar to that which has been adopted previously, and assesses direct overlap between input genes and drug gene signatures. To do so, the Jaccard score, gene overlap fold enrichment, and Fisher’s exact test p values as measures of direct gene overlap are calculated. This analysis is based upon the premise that if disease and drug signatures target similar pathways then they would more than likely have gene overlaps and/or connect extensively in a gene network. Meta-Signature Network Drug Repositioning and ADR Analysis was performed using the multi-tissue network. In Overlap-DR, Jaccard score was used to measure the similarity between the 39 KDG’s gene networks and the drug target gene networks. In Meta-Net-DR, the connectivity of the gene network between drug signatures from PharmOmics and the KDs is used. The z-score of each drug is calculated which represents the distance between the KD network and the PharmOmics drug network. The smaller the z-score, the closer the distance between the networks. The output from these analyses were considered as possible repositioned drugs and were then filtered in Drug Candidate Selection to identify ESCC repositioned drugs.

### Drug candidate selection

Repositioned drugs from both Pharmomics analyses were used as candidates to identify potential drugs for ESCC. Candidate drugs from Overlap-DR results were filtered to keep drugs with an adjusted P-value < 0.05, species equal to *Homo sapiens,* and a within species rank >0. The mean Jaccard score was then calculated and drugs with a Jaccard score less than the mean were removed. Subsequently, the drugs were sorted according to ‘Drug Name’, ‘Within Species Rank’, ‘Jaccard Score’, and ‘P-value’ and duplicate drugs were removed, keeping only the highest-ranking occurrence of each drug. Candidate drugs from Meta-Net-DR were filtered to keep drugs with adjusted p-value < 0.05. Candidate drugs were then sorted according to ‘Drug Name’ and ‘Rank’ and then duplicate drugs were removed, keeping only the highest-ranking occurrence of each drug. The filtered results from Overlap-DR and Meta-Net-DR were then compared to extract candidate drugs common to both arms of analysis. Drugs common to both arms were considered ESCC Repositioned Drugs. ‘Study’, ‘Jaccard Score’, ‘Odds Ratio’, ‘Adj. P-Value’, ‘Within Species Rank’ data from the Overlap-DR analysis and ‘z-score’ from Meta-Net-DR was used to construct the final ESCC Repositioned Drugs table. ESCC Repositioned Drugs were then sorted according to z-score (Table 3).

### Drug candidate validation

In order to validate the repositioned drugs that were identified by the analysis, we performed a literature search to ascertain whether the drugs have previously been used in ESCC treatment (Table 4). Each drug was then investigated using the drug binding database Binding DB. For each ESCC Repositioned Drug, we identified which proteins they display a high binding affinity to. We then performed a literature search on these proteins, using Google Scholar and PubMed, to ascertain whether or not they have previously been shown to be ESCC-related *in vitro* or *in vivo* (Table 5). Finally, we performed a literature search on novel drugs identified by our analysis to elucidate the underlying biological processes and causal mechanisms which would explain why it is predicted to have therapeutic utility (Table 6).

## Data Availability

Publicly available datasets were analyzed in this study. The names of the repository/repositories and accession number(s) can be found in the article/Supplementary Material.

## Supplementary material

Supplementary Figure S1 This figure shows the enriched gene set categories for the Gene Set Enrichment Analysis for Biological Processes.

Supplementary Figure S2 This figure shows the enriched gene set categories for the Gene Set Enrichment Analysis for Cellular Component.

Supplementary Figure S3 This figure shows the enriched gene set categories for the Gene Set Enrichment Analysis for Molecular Function.
