# Supplementary material for: Drug repositioning for esophageal squamous cell carcinoma
Source: Front Genet. 2022 Sep 28;13:991842. doi: 10.3389/fgene.2022.991842 (PMC9554346; doi:10.3389/fgene.2022.991842)
Supplement: Supplementary file 3 [file Table1.DOCX]

**Supplementary File**

**
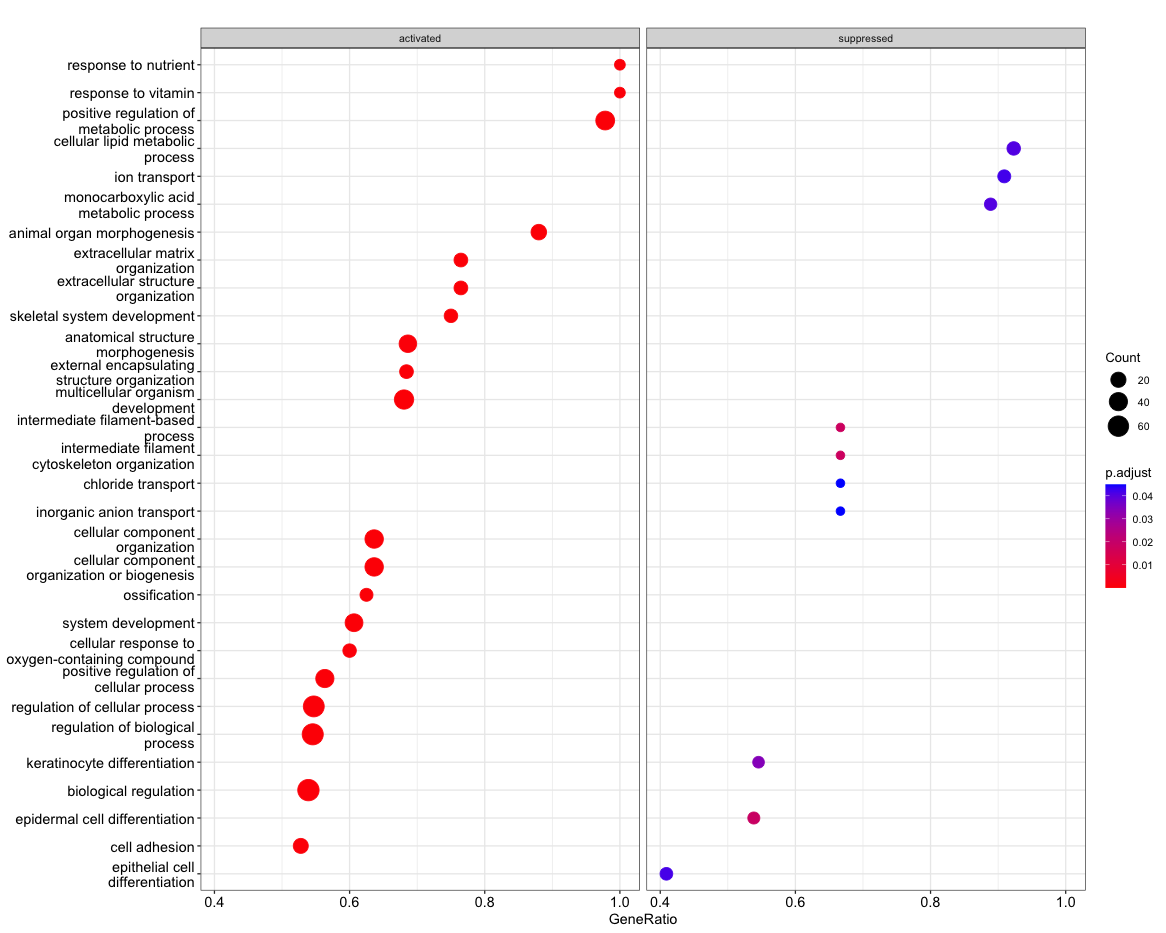
Supplementary Figure 1.** This figure shows the enriched gene set categories for the Gene Set Enrichment Analysis for Biological Processes.

**
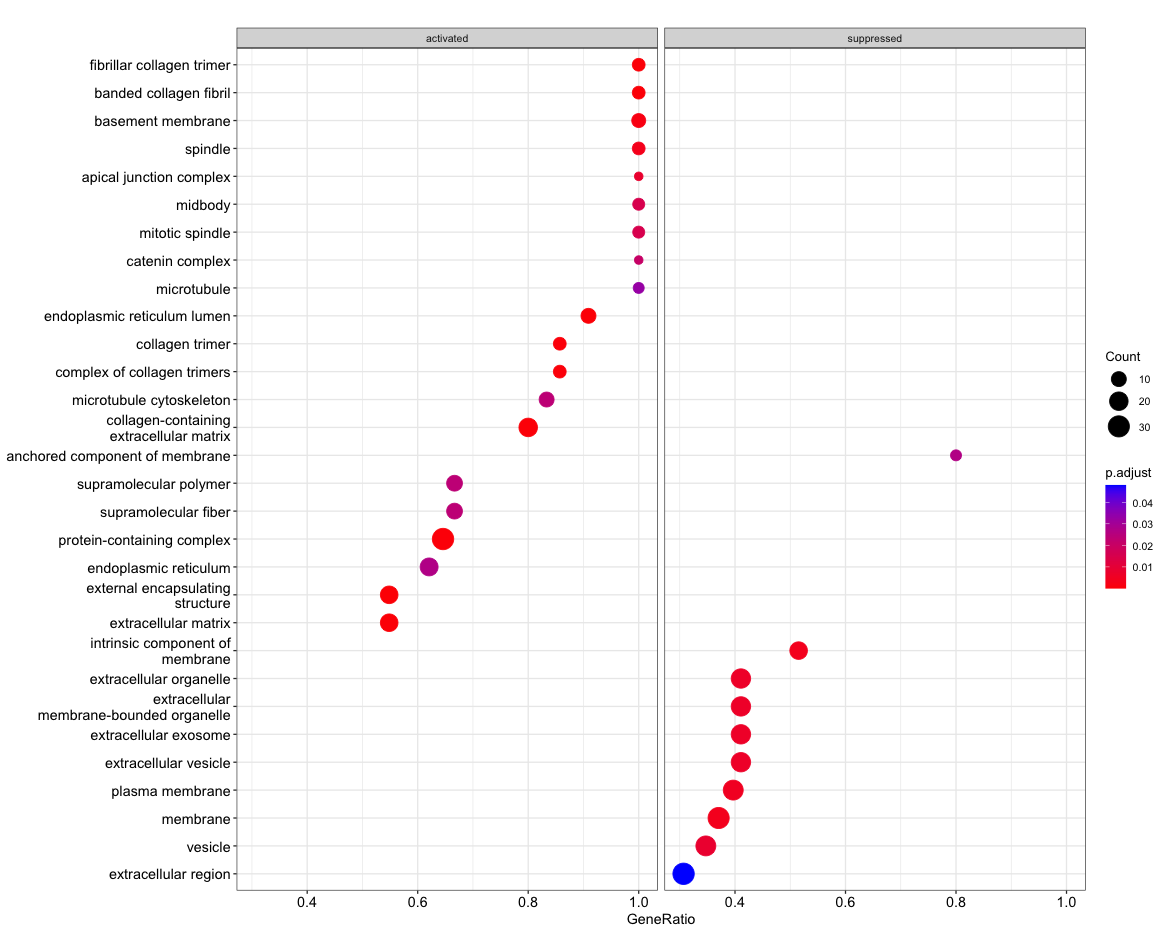
**

**Supplementary Figure 2.** This figure shows the enriched gene set categories for the Gene Set Enrichment Analysis for Cellular Component.

**
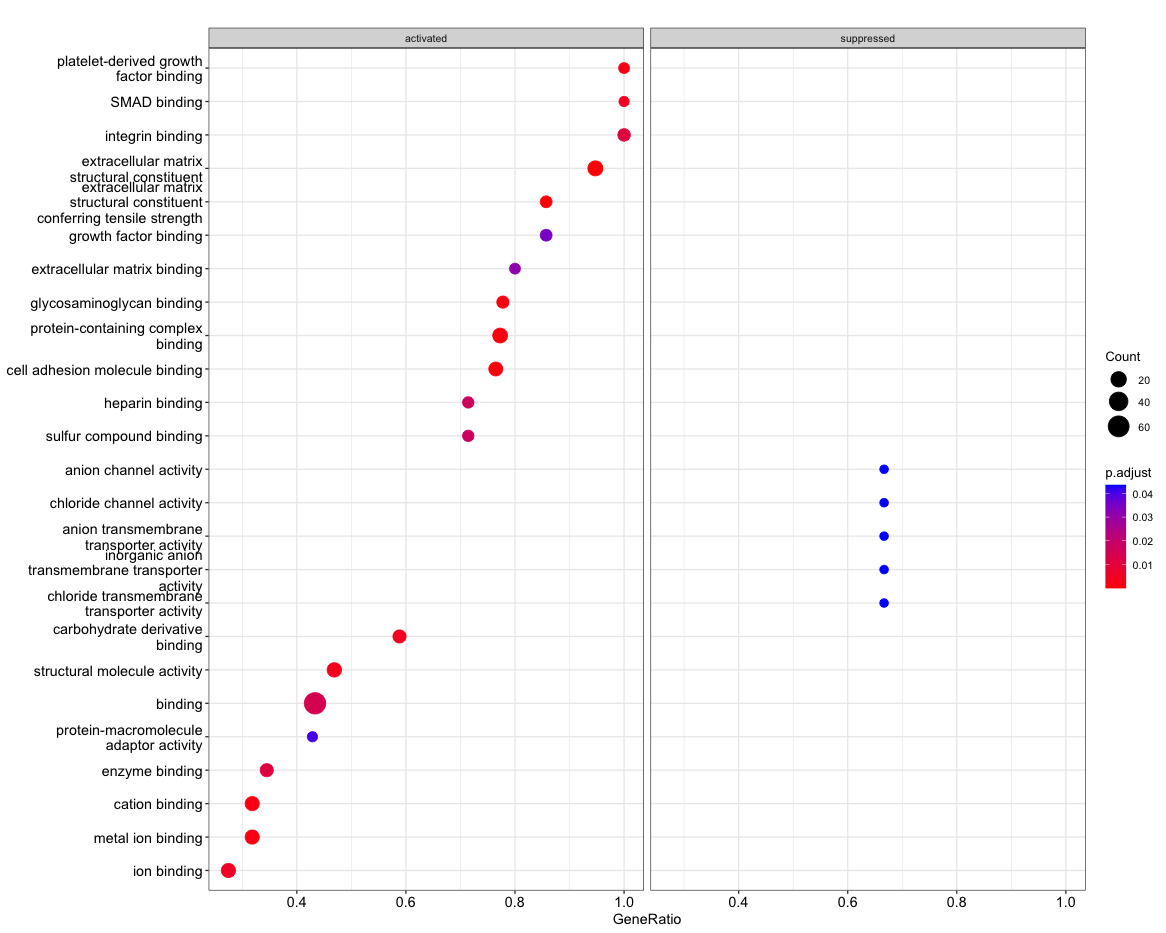
**

**Supplementary Figure 3.** This figure shows the enriched gene set categories for the Gene Set Enrichment Analysis for Molecular Function.
